# *S*-Allylmercaptocysteine Attenuates Cisplatin-Induced Nephrotoxicity through Suppression of Apoptosis, Oxidative Stress, and Inflammation

**DOI:** 10.3390/nu9020166

**Published:** 2017-02-20

**Authors:** Xiaosong Zhu, Xiaoyan Jiang, Ang Li, Zhongxi Zhao, Siying Li

**Affiliations:** 1 School of Pharmaceutical Sciences, Shandong University, 44 West Wenhua Road, Jinan 250012, China; zxs_sdu@163.com (X.Z.); jiangxiaoyan1121@163.com (X.J.); liangliang0725@aliyun.com (A.L.); 2 Shandong Provincial Key Laboratory of Mucosal and Transdermal Drug Delivery Technologies, Shandong Academy of Pharmaceutical Sciences, 989 Xinluo Street, Jinan 250101, China

**Keywords:** cisplatin, SAMC, apoptosis, oxidative stress, inflammation

## Abstract

Cisplatin is a potent chemotherapeutic agent, but its clinical usage is limited by nephrotoxicity. S-allylmercaptocysteine (SAMC), one of the water-soluble organosulfur garlic derivatives, has antioxidant and anti-inflammatory properties and plays an important role in protecting cells from apoptosis. This study aims to examine the protective effects of SAMC on cisplatin nephrotoxicity and to explore the mechanism of its renoprotection. Rats were treated with cisplatin with or without pre-treatment with SAMC. Renal function, histological change, oxidative stress markers and antioxidant enzyme activities were investigated. Apoptotic marker, nuclearfactor (NF)-κB activity, expression of nuclear factor erythroid 2-related factor 2 (Nrf2), NAD(P)H:quinone oxidoreductase 1 (NQO1) and inflammatory cytokines were also examined. The effect of SAMC on cell viability and apoptosis was examined in cultured human kidney (HK-2) cells. SAMC was confirmed to significantly attenuate cisplatin-induced renal damage by using histological pathology and molecular biological method. Pre-treatment with SAMC reduced NF-κB activity, up-regulated Nrf2 and NQO1 expression and down-regulated inflammatory cytokine levels after cisplatin administration. Cisplatin-induced apoptosis in HK-2 cells was significantly attenuated by SAMC. Thus our results suggest that SAMC could be a potential therapeutic agent in the treatment of the cisplatin-induced nephrotoxicity through its anti-apoptotic, anti-oxidant and anti-inflammatory effects.

## 1. Introduction

Cisplatin (cis-diammine-dichloro-platinum, DDP) is a potent anticancer drug commonly used against various types of solid tumors, including breast, ovarian, testicular, head and neck and uterine cervical carcinoma cancers [1]. However, its clinical use is largely limited by serious adverse effects, especially nephrotoxicity [2]. Recent studies have revealed that cisplatin-induced nephrotoxicity is multifactorial and numerous signaling pathways are involved. Oxidative stress, cell apoptosis and inflammation were suggested as important mechanisms in the pathogenesis of cisplatin-induced nephrotoxicity [3,4].

Oxidative stress due to enhanced production of reactive oxygen species (ROS) and impaired antioxidant status has been listed as the main mechanism associated with cisplatin-induced kidney injury [5]. The oxidative condition could stimulate kidney cells to produce a cytoprotective response which acts through the antioxidant response element (ARE) and nuclear factor erythroid 2-related factor 2 (Nrf2). In the nucleus, Nrf2 activates the expression of ARE-driven genes, such as heme oxygenase 1 (HO-1), NAD(P)H:quinone oxidoreductase 1 (NQO1), which alleviate oxidative stress-induced cellular damage [6]. Furthermore, cisplatin could induce apoptosis and necrosis in the proximal tubule [7]. P53 (tumor protein p53), recognized as a major mediator of cisplatin-induced apoptosis, works by transcription-independent mechanisms via interactions with Bcl-2 (B-cell lymphoma 2) family proteins [8]. Moreover, evidence suggests that multipleinflammatory mediators are involved in the pathogenesis of cisplatin nephrotoxicity. These mediators include tumor necrosis factor (TNF)-α, interleukin (IL)-1β, nuclear factor-κB (NF-κB), cyclooxygenase-2 (COX-2), and transforming growth factor-β (TGF-β) [9].

The use of natural medicines in the prevention and treatment of kidney injury is receiving increasing attention [10]. Garlic (*Allium sativum*), a widely used herbal vegetable, has been suggested as an anticancer agent for several decades. It is also used to treat and prevent inflammatory diseases [11]. The beneficial effects of garlic may be attributed to its organosulfur compounds. S-allylmercaptocysteine (SAMC) is a water-soluble sulfur compound present in aged garlic extract (AGE) [12]. Previous studies have shown that SAMC is not only effective in inhibiting cell growth and inducing apoptosis of various cancer cells, but influential in oxidative stress and inflammation [13,14]. Therefore, it has been assumed that SAMC might be responsible for the protective effect of AGE in several experimental models associated with oxidative stress [12,15]. Moreover, a previous study has indicated that SAMC treatment could attenuate gentamicin-induced oxidative and nitrosative stress and renal damage in vivo [14].However, as far as we know, little information about the effect of SAMC on cisplatin-induced nephrotoxicity is available. The aim of the current study was to investigate the potential protective effect of SAMC against cisplatin-induced nephrotoxicity both in vitro and in vivoand to elucidate the underlying molecular mechanisms.

## 2. Materials and Methods

### 2.1. Reagents

SAMC (purity of 96%) was synthesized and purified in our laboratory with a modified procedure as previously reported [16]. SAMC was freshly prepared as a stock solution in phosphate-buffered saline (PBS) for the in vitro assay and was suspended in phosphate-buffered saline containing 10% l-dextrose and 1% gum Arabic (*w/v*; Sigma Chemicals., St Louis, MO, USA) at pH 4.5 for application in rats. Cisplatin was purchased from Sigma Chemicals (St Louis, MO, USA) and was suspended in normal saline. Commercial assay kits for blood urea nitrogen (BUN), creatinine (CRE), glutathione (GSH), superoxide dismutase (SOD), catalase (CAT), and malondialdehyde (MDA) were purchased from Nanjing Jiancheng Bioengineering Research Institute (Nanjing, China). Hematoxylin and eosin (H&E) dye kits were acquired from Nanjing Jiancheng Bioengineering Research Institute (Nanjing, China). Sulphorhodamine B (SRB) was purchased from Sigma Chemicals. DAPI (4′6-diamidino-2-phenylindole) dye kit was obtained from Beijing Solarbio Co., Ltd. (Beijing, China). Anti-NF-κBp65, anti-COX-2, anti-cleaved PARP (poly ADP ribose polymerase) antibodies were provided by Cell Signaling Technology (Danvers, MA, USA). Anti-Bcl-2, anti-p53, anti-cytochrome c, anti-TGFβ1, and anti-NQO1 antibodies were provided by Abcam (Cambridge, MA, USA). Anti-IκBα (inhibitor of NF-κB-alpha) antibodies were provided by Bioss (Beijing, China). Anti-Nrf2 antibodies were provided by Santa Cruz Biotechnology (Santa Cruz, CA, USA). Secondary antibodies were provided by ZSGB-BIO (Beijing, China). Deoxynucleotidyl transferase-mediated uridine triphosphate (dUTP) nick-end labelling (TUNEL) apoptosis detection kit was provided by Roche Applied Science.

### 2.2. Cell Culture and Drug Treatment

The immortalized proximal tubule epithelial cell line from the normal adult human kidney (HK-2) was purchased from Cell Bank of China (Shanghai, China) and cultured. Briefly, cells were passaged every 2–3 days in 100-mm dishes (Corning, NY, USA) using Dulbecco’s modified Eagle’s medium (DMEM)-F12 (HyClone, Logan, UT, USA) supplemented with 10% fetal bovine serum (FBS; BI, Cromwell, CT, USA), 100 units/mL penicillin and 100 mg/mL streptomycin (Solarbio, China). These cells were incubated in a humidified atmosphere of 5% CO_2_, 95% air at 37 °C for 24 h and subcultured at 70%–80% confluence. For the drug treatments, HK-2 cells were seeded at 5 × 10^4^ cells/mL and treated with different concentrations of cisplatin for the periods of time. Pretreatment of SAMC was carried out 4 h prior to the application of cisplatin.

### 2.3. Cell Viability Assay

Cell viability was determined using the SRB (Sulphorhodamine B) assay [17]. HK-2 cells were seeded in 96-well tissue culture microplates at 5 × 10^4^ cells/well for 24 h, and then the cells were incubated with different treatments for different periods of time. The treated cells were then fixed with 10% trichloroacetic acid (TCA) for 1 h at 4 °C, the 96-well plates were washed three times with distilled water and allowed to dry in the air. Each well was added with 50 μL of SRB solution and stained for 15 min at room temperature. The SRB staining solution was removed by washing the plates quickly with 1% (*v/v*) acetic acid three times, and the plates were dried in the air. The dried materials in each well were solubilized by adding 200 μL of 10 mM unbuffered Tris Base (pH 10.5). The cell viability was detected by measuring the absorbance at 540 nm on a plate reader (TECAN, Lyon, France).

### 2.4. DAPI Staining

DNA fragmentation during cell apoptosis can be detected by DAPI staining. Treated HK-2 cells cultured on cover glasses in 12-well plates were washed with PBS and fixed with cold methanol/acetone (1:1, stored at −20 °C) for 5 min at room temperature. The solution was removed and washed with PBS, and then incubated with the DAPI solution for 10 min at room temperature. Fluorescent cells were observed under a fluorescence microscope (Olympus, Tokyo, Japan).

### 2.5. Flow Cytometric Analysis

The cell cycle distribution was analyzed by flow cytometry. HK-2 cells were trypsinized after treatment, washed with PBS and fixed in 70% ethanol overnight at −20 °C. Next, cells were incubated with RNase (Ribonuclease) and stained with propidium iodide (50 μg/mL; Sigma) for 30 min. Cell cycle distribution was determined using a Cytomics FC500 Flow Cytometer (Beckman Coulter, CA, USA). The percentages of cells in G1, G2/M, and S phases were analyzed by using ModFit LT software 3.0 (Varity Software House, Topsham, MA, USA), including the sub-G1 phase of apoptotic cells.

### 2.6. Animals and Drug Treatment

Male Sprague–Dawley rats (Institute of Laboratory Animal Sciences, Beijing, China) were given a standard laboratory diet and water, and were cared for under a protocol approved by the Institutional Animal Care and Use Committee of the Shandong University. At the start of the experiments, the rats, weighing 160–180 g were kept at standard housing facilities (24 ± 1 °C, 45% ± 5% humidity and 12 h light/dark cycle). They were supplied with standard laboratory chow and water and left to acclimatize for one week before the experiment. The animals were randomly divided into five equal groups in separate plastic cages, six rats each. Group I (Vehicle control, Control), animals were administered with phosphate-buffered saline containing 10% l-dextrose and 1% gum Arabic (*w/v*) intraperitoneally once daily for 20 consecutive days and a single intraperitoneal injection of normal saline on the 15th day. In group II (Cisplatin control), animals were administered phosphate-buffered saline containing 10% l-dextrose and 1% gum Arabic (*w/v*) intraperitoneally once daily for 20 consecutive days and a single intraperitoneal injection of cisplatin (7 mg/kg dissolved in normal saline) on the 15th day. Animals were intraperitoneally administered SAMC of 10 mg/kg (group III), 20 mg/kg (group IV), and 30 mg/kg (group V) once daily for 20 consecutive days and a single intraperitoneal injection of cisplatin (7 mg/kg dissolved in normal saline) on the 15th day. At the end of the experiment (i.e., on the 20th day), the body weight of all animals was recorded. Blood samples were collected from all the experimental animals and serum was separated. The animals were anaesthetized by an intraperitoneal injection of 10% chloralic hydras (3.5 mL/kg) and euthanized by cervical dislocation; kidney tissues were isolated, relative weights of kidneys (i.e., kidney to body weight ratio normalized to 100 g body weight of animals) were determined and then stored at −80 °C for further study.

### 2.7. Renal Function Monitoring

On the day of the sacrifice, blood was collected immediately. Blood urea nitrogen and creatinine levels in blood were determined by commercially available diagnostic kits according to the manufacturer’s instructions.

### 2.8. Measurement of Oxidative Stress Markers and Antioxidant Enzymes Activities

A 10% kidney-tissue homogenate was prepared with phosphate buffer saline (50 mM, pH 7.4) using Teflon homogenizer. A part of the homogenate was mixed with an equal volume of 10% trichloroacetic acid and centrifuged at 3000 rpm for 15 min at 4 °C. Its supernatant was used to determine the content of GSH and MDA (oxidative stress markers) enzymes. The remaining part of the homogenate was centrifuged at 12,000× *g* for 45 min at 4 °C and the supernatant was used for estimation of the antioxidant enzyme activities of SOD and CAT enzymes. Activities of GSH, MDA, SOD and CAT were measured using a commercial kit according to the manufacturer’s instructions.

### 2.9. Measurement of Kidney TNF-α and IL-1β Levels

A 10% kidney-tissue homogenate was prepared with phosphate buffered saline (50 mM, pH 7.4) using Teflon homogenizer. Then the homogenates were centrifuged at 3000× *g* for 10 min. The supernatants were kept at −80 °C till measurement. The levels of TNF-α and IL-1β in kidney tissues were measured using commercial ELISA kits (MULTI SCIENCES, Hangzhou, China) according to the manufacturer’s protocols.

### 2.10. Detection of Apoptosis

Apoptosis was assessed by terminal deoxynucleotidyl transferase-mediated uridine triphosphate (dUTP) nick-end labelling (TUNEL) using an in situ cell death detection kit. The serial 4-μm sections were cut from formalin-fixed kidney tissues and the staining conducted according to the manufacturer’s instructions. Cells with brown-stained nuclei were considered TUNEL positive [18]. The proportion of positive cells was examined under a morphometric microscope.

### 2.11. Histopathological Examination and Immunohistochemistry for Detection of NF-κB

The kidney tissues underwent hematoxylin and eosin staining. Briefly, freshly dissected tissues were fixed and embedded in paraffin. After being cut into 4-μm slices, the sections were deparaffinized and stained with hematoxylin and eosin solution.

Detection of NF-κB was performed according to Pedrycz and Czerny [19]. Briefly, anti-NF-κB p65 antibody was 1:800 diluted and immunostaining was done following a standard protocol of the DAB Substrate Kit. Finally, the sections were counterstained with hematoxylin, dehydrated, and mounted for imaging.

### 2.12. Western Blot Analysis

Western blot analysis was performed as previously described [20]. In their vitro assay, treated HK-2 cells were harvested and the lysates were fractionated using 10% sodium dodecyl sulfate-polyacrylamide gel electrophoresis (SDS-PAGE). The rat kidney tissues were dispersed in PBS mechanically. The supernatant liquids were then collected and the total proteins were determined. Cell lysates were separated using 10% SDS-PAGE and electro-transferred onto the polyvinylidene difluoride (PVDF) membrane. Their expression levels were determined using primary antibodies. The PVDF membranes were washed in Trisbuffered saline (TBS) containing 0.1% Tween-20 (TBST) and incubated with appropriate secondary antibodies. Bound antibodies were visualized by an enhanced chemiluminescence reagent (Millipore) and the images were captured by Alphalmager HP system (Cell biosciences, Wallingford, CT, USA).

### 2.13. Statistical Analysis

The data were described as the mean ± SEM (standard error of the mean) and analyzed using ANOVA. Differences with *p* values < 0.05 were considered statistically significant. Statistical analysis was performed with SPSS/Win13.0 software (SPSS, Inc., Chicago, IL, USA).

## 3. Results

### 3.1. SAMCAttenuates Cisplatin-Induced Cytotoxicity in HK-2 Cells

**Figure 1:**
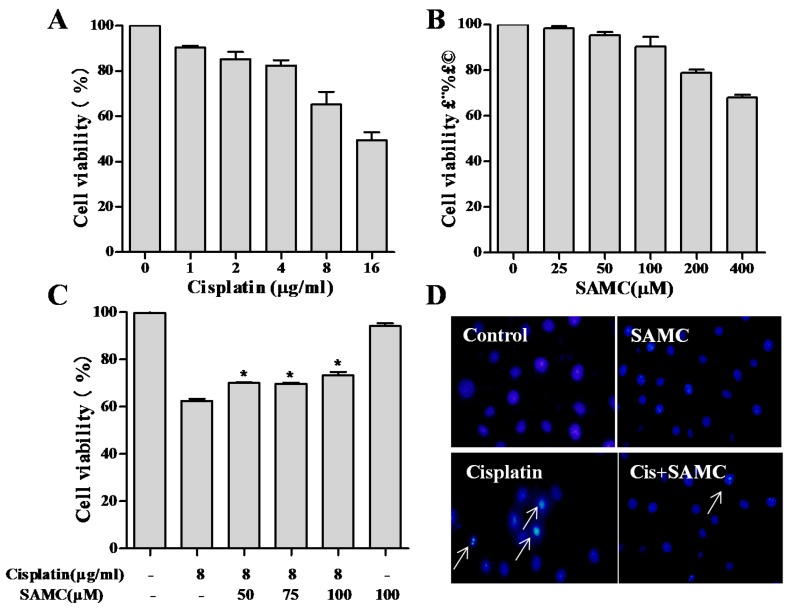
S-allylmercaptocysteine (SAMC) attenuated cisplatin-induced cytotoxicity in human kidney (HK-2) cells. Cell viability was assessed using the SRB (sulphorhodamine B) assay. HK-2 cells were treated with various concentrations of cisplatin (**A**), SAMC (**B**), Cells were pretreated with or without SAMC (50–100 μM) for 4 h and then cultured in the presence or absence of 8 μg/mL cisplatin for 24 h (**C**,**D**) HK-2 cells treated with 8 μg/mL cisplatin and 8 μg/mL cisplatin plus 100 μM SAMC were stained with DAPI (4’6-diamidino-2-phenylindole) to investigate apoptotic-like nuclear morphology. White arrows indicate condensed nuclei. All data were obtained from three independent experiments and presented as the means ± SEM (standard error of the mean). Bars with different characters are statistically different at * *p* < 0.05 level.

Tubular cell injury and death have been regarded as the main pathophysiological activities of cisplatin nephrotoxicity [21]. We examined the cytotoxic effects of cisplatin and possible protective effects of SAMC on proximal tubularHK-2 cells by using SRB assay. As shown in Figure 1, 8 μg/mL cisplatin with 35% cell death was selected for further study. For SAMC, 50, 75 and 100 μM were chosen as the concentration in the conventional culture medium because of their insignificant effect on the growth rate and cell morphology of HK-2 cells. Cell viability was significantly increased by co-treatment with SAMC in a dose-dependent manner compared with the cisplatin alone group (Figure 1C).

### 3.2. SAMCRescues HK-2 Cells from Cisplatin-Induced Apoptosis 

**Figure 2:**
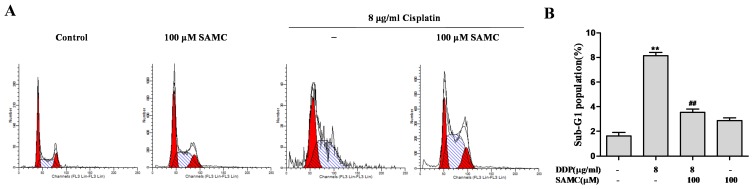
SAMC rescued HK-2 cells from cisplatin-induced apoptosis. (**A**,**B**) SAMC decreased cisplatin-induced apoptotic cell death. Cells were pretreated with or without 100 μM SAMC for 4 h and then cultured in the presence or absence of 8 μg/mL cisplatin for 24 h. ** *p* < 0.01 vs. control, ^##^ *p* < 0.01 vs. cisplatin.

Apoptotic pathway is reported to be a molecular mechanism of cisplatin-induced nephrotoxicity. We next examined the protective effects of SAMC on cisplatin-induced apoptosis by DAPI staining assay. After treatment with cisplatin, HK-2 cells exhibited typical apoptotic features, such as nuclear fragmentation, as evidenced by the arrows in Figure 1D. However, co-treatment with SAMC effectively prevented these cisplatin-induced morphological changes. By contrast, control and SAMC-treated cells showed normal nuclei shape. The protection of SAMC on HK-2 cells against cisplatin was further confirmed using flow cytometric analysis. Data in Figure 2 showed that cells treated with 100 μM SAMC alone nearly did not change the cell cycle distribution of HK-2 cells, while the cisplatin-treated group induced an increase in sub-G1 cell population, which reflected the proportion of apoptotic cells. By co-treatment with SAMC, the sub-G1 peak was significantly decreased (*p* < 0.01).

**Figure 3:**
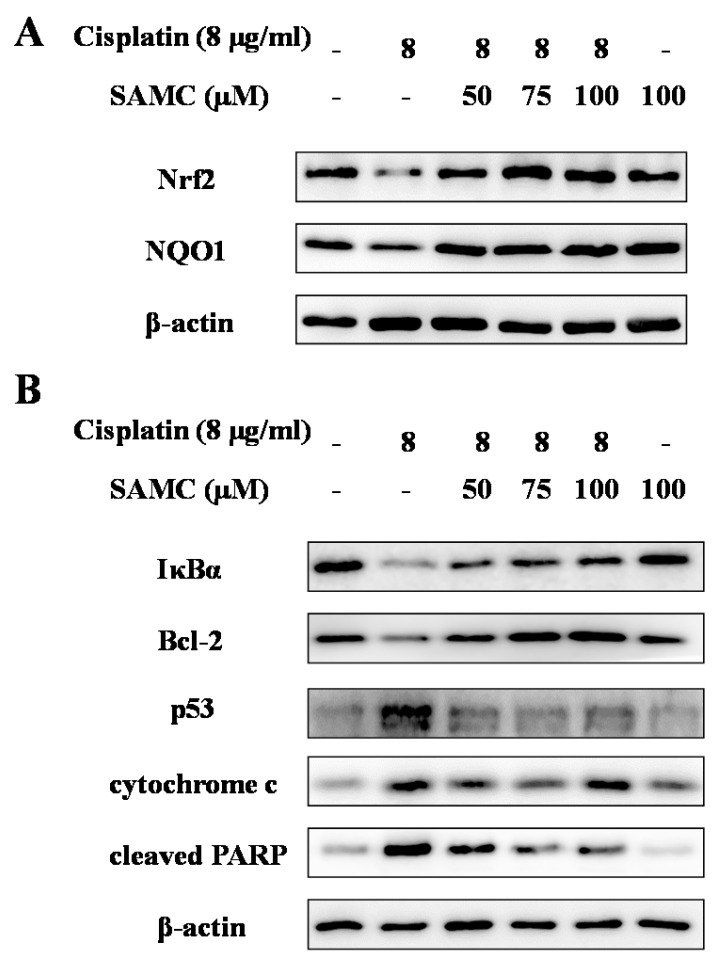
Effects of SAMC on nuclear factor erythroid 2-related factor 2 (Nrf2) and NAD(P)H:quinone oxidoreductase 1 (NQO1) expression (**A**); NF-κB activity and the expression of apoptosis-related proteins (**B**) in HK-2 cells. Cells were pretreated with or without SAMC (50–100 μM) for 4 h and then cultured in the presence or absence of 8 μg/mL cisplatin for 24 h. The expression levels of Nrf2, NQO1, IκBα, Bcl-2, p53, cytochrome c and cleaved PARP (poly ADP ribose polymerase) were analyzed by Western blot. Equal protein loading was confirmed by analysis of β-actin in the protein extracts.

The signaling pathways that lead to cisplatin-induced tubular cell apoptosis are complex and remain to be elucidated. To investigate the underlying mechanisms for the protection of SAMC against cisplatin-induced apoptosis, we analyzed various apoptosis related proteins in HK-2 cells. As shown in Figure 3B, the expression levels of p53 and cytochrome c were markedly up-regulated, while the expression of Bcl-2 was down-regulated after cisplatin treatment. PARP serves as a marker of apoptosis, which has been found as a DNA repair enzyme in the apoptosis pathways. In this study, cisplatin increased the expression of cleaved PARP in HK-2 cells. However, these changes were recovered by co-treatment with SAMC, indicating that SAMC can reduce cisplatin-induced apoptosis through modulation of p53 pathway and Bcl-2 family proteins.

### 3.3. Effect of SAMC on Cisplatin-Induced Renal Injury Parameters In Vivo

**Figure 4:**
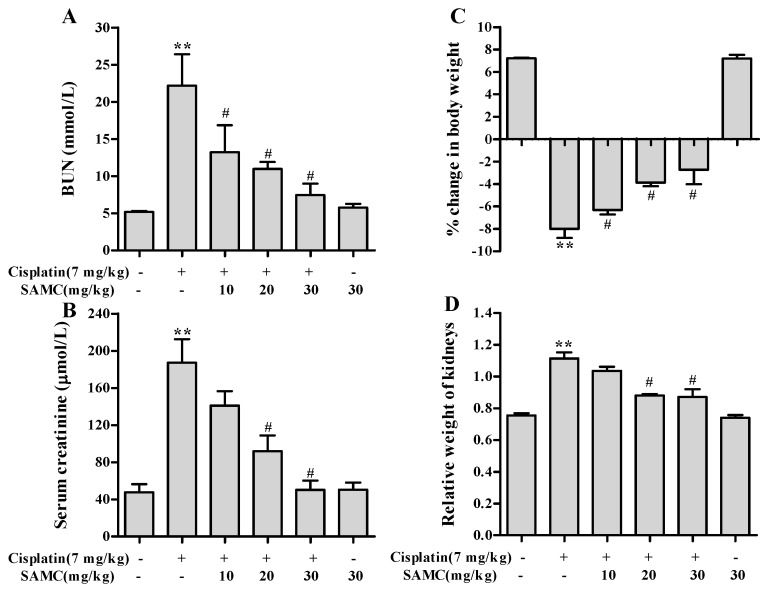
Effect of SAMC on cisplatin-induced changes in serum renal function parameters. (**A**) Blood urea nitrogen (BUN); (**B**) Creatinine; (**C**) Percentage change in body weight and (**D**) Relative weight of kidneys in different experimental groups. SAMC was administered intraperitoneally at three different doses i.e., 10 mg/kg, 20 mg/kg and 30 mg/kg for 20 consecutive days and a single dose of cisplatin (7 mg/kg, intraperitoneally) was administered on the 15th day. On the 20th day, serum levels of BUN and creatinine, percentage change in body weight and relative weight of kidneys were recorded. Values are expressed as mean ± SEM (*n* = 6). ** *p*< 0.01 vs. control, ^#^ *p*< 0.05 vs. cisplatin.

Cisplatin caused severe renal dysfunction as shown in parameters BUN and creatinine (Figure 4). Cisplatin administration resulted in 4.3- and 3.9-fold increase in BUN and creatinine respectively. SAMC attenuated both kidney injury markers in a dose-dependent manner. No mortality was observed in animals treated with cisplatin and/or SAMC during the study period. For calculation of percentage of body weight gain/loss, the day of cisplatin administration was considered as day 0. We observed a 7.2% body weight gain in vehicle control group and an 8.5% body weight loss in cisplatin group. SAMC treatment dose-dependently attenuated the body weight loss induced by cisplatin (Figure 4C). Similarly, the relative weight of kidneys was elevated by 1.5-fold in cisplatin alone-treated rats compared with vehicle control group. This change was prevented by SAMC at doses 20 and 30 mg/kg (*p* < 0.05) (Figure 4D). Treatment with SAMC alone did not show any significant effects on these parameters.

### 3.4. SAMC Ameliorates Tubular Necrosis and Apoptosisin Cisplatin-Induced Renal Injury

**Figure 5:**
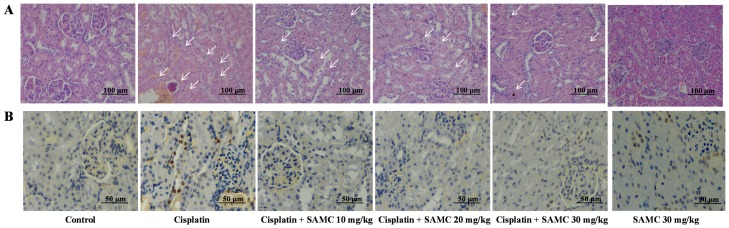
Effect of SAMC on renal histology (**A**) and tubular apoptosis (**B**) in cisplatin-induced renal injury in rats. White arrows show tubular damage.

Histochemical examination revealed necrosis and vacuolation of epithelial cells in renal tubules after cisplatin treatment as shown by the arrows in Figure 5A. Fortunately, pretreatment with SAMC dose-dependently prevented the renal damage, suggesting that SAMC may protect against cisplatin-induced renal injury. Renal tubular epithelial cell apoptosis was quantified by TUNEL. Kidneys from cisplatin-treated group showed nuclear changes consistent with apoptotic cell death. Pretreatment with SAMC reduced the extent of apoptotic cell death caused by cisplatin (Figure 5B).

### 3.5. SAMC Attenuates Cisplatin-Induced Oxidative Stress and BoostsRenal Antioxidant Defenses

**Figure 6:**
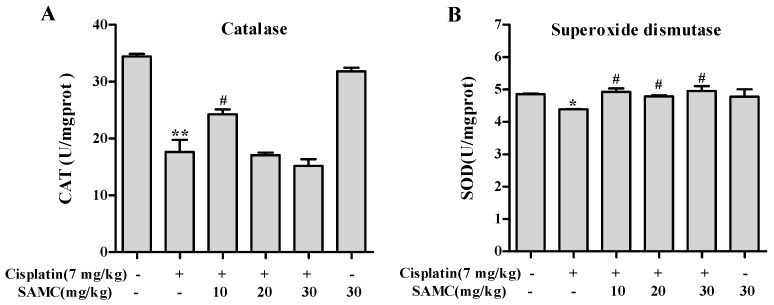
Effect of SAMC on cisplatin-induced changes in antioxidant enzymes activities in renal tissues of rats. (**A**) Catalase; (**B**) Superoxide dismutase. SAMC was administered intraperitoneally at three different doses, i.e., 10 mg/kg, 20 mg/kg and 30 mg/kg for 20 consecutive days and a single dose of cisplatin (7 mg/kg, i.p) was administered on the 15th day. Values are expressed as mean ± SEM (*n* = 6). * *p* < 0.05 vs. control, ** *p* < 0.01 vs. control, ^#^ *p* < 0.05 vs. cisplatin.

**Figure 7:**
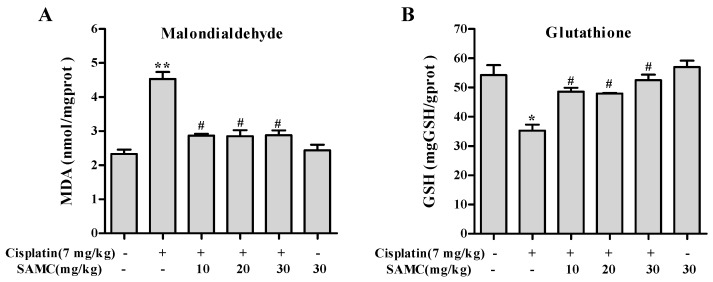
Effect of SAMC on cisplatin-induced changes in oxidative stress markers in renal tissues of rats. (**A**) Malondialdehyde; (**B**) Glutathione. SAMC was administered intraperitoneally at three different doses, i.e., 10 mg/kg, 20 mg/kg and 30 mg/kg for 20 consecutive days and a single dose of cisplatin (7 mg/kg, i.p) was administered on the 15th day. Values are expressed as mean ± SEM (*n* = 6). * *p* < 0.05 vs. control, ** *p* < 0.01 vs. control, ^#^ *p* < 0.05 vs. cisplatin.

**Figure 8:**
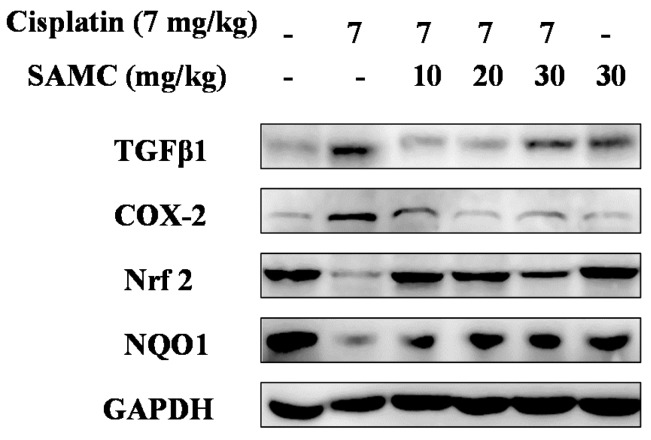
Effect of SAMC on cisplatin-induced changes on the expression of proteins in renal tissues of rats. SAMC was administered intraperitoneally at three different doses, i.e., 10 mg/kg, 20 mg/kg and 30 mg/kg for 20 consecutive days and a single dose of cisplatin (7 mg/kg, i.p) was administered on the 15th day. The expression levels of transforming growth factor-β (TGFβ1), cyclooxygenase-2 (COX-2), Nrf2 and NQO1 were analyzed by Western blot. Equal protein loading was confirmed by analysis of GAPDH (glyceraldehyde 3-phosphate dehydrogenase) in the protein extracts.

Oxidative stress is considered to be one of the main mechanisms of cisplatin-induced acute organ injury [5]. As shown in Figure 6, cisplatin exposure resulted in notable decrease of SOD (*p* < 0.05) and CAT (*p* < 0.01) activity compared with the control group and SAMC-alone group. In contrast, treatment with SAMC improved SOD activity to normal level. Only SAMC at 10 mg/kg produced a significant increase (*p* < 0.05) in CAT activity. These data suggest that SAMC alleviated oxidative injury in kidney via up-regulating antioxidant enzyme activity. Concomitantly, rats injected with cisplatin showed a remarkable elevation of MDA (*p* < 0.01) and depletion of GSH (*p* < 0.05) (Figure 7). Administration of SAMC decreased MDA levels and restored the antioxidant status as demonstrated by an increase in GSH content, indicating that SAMC protects the kidneys from cisplatin-induced oxidative stress. Nrf2 plays an important role in regulating antioxidant responses [22]. To study whether the protective effect of SAMC was mediated by Nrf2 signaling pathway, we investigated the expression of Nrf2 and NQO1 both in vitro and in vivo. The results showed that treatment of cisplatin down-regulated the expression of Nrf2 and NQO1. In contrast, treatment with SAMC could up-regulate the protein level of Nrf2 and NQO1 (Figure 3A and Figure 8).

### 3.6. SAMCSuppresses the Inflammatory Events in Cisplatin-Induced Nephrotoxicity

**Figure 9:**
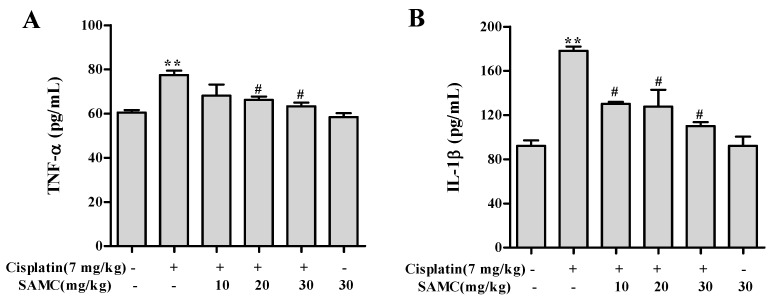
Effect of SAMC on the levels of renal inflammatory cytokines in cisplatin-induced nephrotoxicity. SAMC was administered intraperitoneally at three different doses, i.e., 10 mg/kg, 20 mg/kg and 30 mg/kg for 20 consecutive days and a single dose of cisplatin (7 mg/kg, i.p) was administered on the 15th day. On 20th day, serum levels of tumor necrosis factor (TNF)-α (**A**) and interleukin (IL)-1β (**B**) were recorded. Values are expressed as mean ± SEM (*n* = 6). ** *p* < 0.01 vs. control, ^#^ *p* < 0.05 vs. cisplatin.

Inflammation is involved in thepathogenesis of cisplatin nephrotoxicity [23]. In order to evaluate whether SAMC was able to attenuate the cisplatin induced renal inflammation, we analyzed the inflammatory cytokine levels in vivo. As shown in Figure 8 and Figure 9, cisplatin injection caused higher levels of TNF-α and IL-1β in serum and higher protein levels of TGFβ1 and COX-2 in kidney tissues than those in normal group. In contrast, pretreatment with SAMC significantly suppressed the production of these inflammatory cytokines.

**Figure 10:**
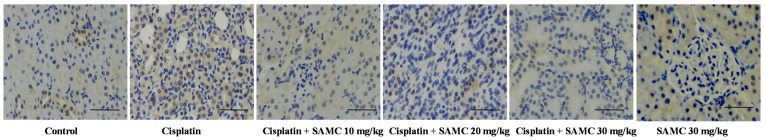
Effect of SAMC on nuclear factor NF-κB expression in cisplatin-induced renal injury in rats. Bars indicate 50 μm.

NF-κB plays a key role in oxidative stress, inflammation and mitochondrial gene regulation. To further investigate the mechanism of SAMC on cisplatin induced renal inflammation, we analyzed the protein expression of NF-κB (p65) in kidney tissues. As shown in Figure 10, the NF-κB activity was markedly induced in renal tubular cells of cisplatin-treated rats compared with the control group and SAMC alone group. Correspondingly, the increased activity of NF-κB was significantly inhibited by pretreatment with SAMC. Moreover, western blot analysis showed that the levels of IκBα protein in HK-2 cells were significantly down-regulated after cisplatin treatment. Pretreatment with SAMC enhanced the IκBα protein level in HK-2 cells in a dose-dependent manner (Figure 3).

## 4. Discussion

Cisplatin is one of the most important chemotherapeutic agents in chemotherapy whose usage is limited largely by nephrotoxicity. It has been reported that cisplatin-induced nephrotoxicity could be attributed to oxidative stress and inflammation [21]. Garlic and its compounds exert strong biological activities including anti-oxidative, anti-inflammatory and anti-cancer activities [11]. Although a recent study reported theprotective effects of aged garlic extract (mainly contain allicin, *S*-Allylcysteine and *S*-allylmercaptocysteine) on cisplatin-induced nephrotoxicity in rats [24], the efficacy and relative mechanism of SAMC itself on cisplatin-induced nephrotoxicity has not been reported so far. In the present study, we demonstrated the protective effects of SAMC on cisplatin-induced nephrotoxicity and the underlying mechanisms in vitro and in vivo for the first time. Our results showed that SAMC treatment reduced cisplatin-induced apoptosis and oxidative stress, restored antioxidant enzyme activities and attenuated expressions of apoptosis, oxidative stress and inflammation related proteins, thus forming the molecular basis for protective mechanism of SAMC against cisplatin-induced nephrotoxicity.

The human kidney epithelial HK-2 cells have been shown to retain morphological and biochemical characteristics of human renal proximal tubular cells, providing an appropriate in vitro experimental system to evaluate cisplatin-induced nephrotoxicity. The data from the present study showed that pretreatment with SAMC improved HK-2 viability in cells treated with 8 μg/mL cisplatin. The demonstration of lower cell survival benefit from SAMC with low (6 μg/mL) and high (10 μg/mL) cisplatin exposures suggests a potential targeted role under conditions of medium cisplatin (8 μg/mL) exposures (data not shown).

In vivo, rats treated with cisplatin showed significant decrease in body weight and increase in the relative weight of the kidneys. Cisplatin-induced body weight loss might be due to gastrointestinal toxicity and subsequent decrease in appetite [24]. Pretreatment with SAMC alleviated the severe decrease in body weight, giving an approval on the protective effect of SAMC against the toxicity of cisplatin in renal tissues. The increase in relative kidney weight might be due to the edema of renal parenchyma since cisplatin could cause renal inflammation [24]. This increase was inhibited by SAMC, which could be attributed to its anti-inflammatory effect [25]. Taken together, SAMC could be a promising candidate for clinical use in patients undergoing treatment with cisplatin to ameliorate the toxicity.

Apoptosis was a predominant cause of cisplatin-induced nephrotoxicity. Tumor suppressor p53 plays a critical role in the induction of apoptosis. Activation of p53 could mediate the up-regulation of Bax (B-cell associated X protein), down-regulation of Bcl-2, and PARP cleavage [8]. The release of mitochondrial cytochrome c into the cytosol occurs with PARP degradation [26]. In agreement with these studies, our results showed that cisplatin activated p53, causing the reduced expression ofBcl-2 and inducing degradation of PARP to promote apoptotic activity in HK-2 cells. In addition, we found that cisplatin induced cytochrome c release, thereby triggering the mitochondrial apoptotic pathway. Cisplatin administration is known to be associated with increased formation of free radicals, heavy oxidative stress and lipid peroxidation. Previous studies have indicated that cisplatin could induce oxidative damage and lipid peroxidation in kidney tissues [27,28]. In our study, the level of MDA, an important marker of oxidative stress, increased in the cisplatin-intoxicated rats, resulting in consumption of glutathione and other endogenous antioxidants such as SOD and CAT. However, pretreatment with SAMC maintained glutathione and other antioxidants in renal tissue. Our observations also gain support by a study in which it was shown that SAMC attenuated gentamicin-induced oxidative stress and renal damage in rats cells through its anti-oxidant effect [14]. Nrf2 is a key transcriptional factor that activates the antioxidant-reactive element (ARE) and ARE-driven genes, including HO-1 and NQO1 [6]. Consistent with results from other studies, the present study showed that Nrf2 and NQO1 expression in the cisplatin-induced group was lower than those of controls, whereas SAMC treatment induced activation of Nrf2 and NQO1. Similar to our results, a previous study reported that S-allylcysteine (SAC) could protect against cisplatin-induced nephrotoxicity in rats by activating the Nrf2 pathway [29].

A growing body of evidence suggests that inflammation is associated with cisplatin-induced nephrotoxicity [9,30]. Previous studies showed that cisplatin activates the NF-κB pathway depending on the degradation of IκB, which could increase a series of inflammatory cytokines including TNF-α, IL-1β and TGFβ1 [31]. Pro-inflammatory cytokines could in turn initiate the degradation of IκB (inhibitor of NF-κB) [32]. Since NF-κB is the main regulator of molecules involved in inflammation and apoptosis, the inhibition of NF-κB by SAMC might be a critical step for the prevention of inflammatory response and oxidative stress injury in cisplatin-induced nephrotoxicity. In the present study, the SAMC-pretreated group showed declined expression of NF-κB and increased expression of IκB. It has been reported that renal COX-2 expression increases concomitantly with kidney injury, renal inflammation and oxidative stress in rats treated with cisplatin [33]. In our study, SAMC was shown to markedly suppress the cisplatin-induced elevation in inflammation cytokines including TNF-α, IL-1β, TGFβ1 and COX-2; clearly indicating that SAMC exerted an anti-inflammatory effect in cisplatin-induced renal injury. Interestingly, the results of the current study are consistent with a previous report that SAMC prevented liver disease by diminishing NF-κB activity andinhibiting the expression of pro-inflammatory cytokinesTNF-α, IL-1β and TGFβ1, as well as inflammatoryenzymes COX-2 and iNOS (inducible nitric oxide synthase) [25].

## 5. Conclusions

In conclusion, the present study demonstrated the protective effect of SAMC against cisplatin-induced nephrotoxicity in rats and cisplatin-induced cytotoxicity in HK-2 proximal tubular cells. The renoprotective effect of SAMC could be due to the reduction of oxidative stress, restoration of antioxidant enzyme activities and suppression of apoptosis. In addition, the mechanism of this renoprotective effect may also involve inhibition of NF-κB activation and attenuation of pro-inflammatory mediators. However, further studies are needed to evaluate the potential use of SAMC as an adjuvant therapy to attenuate cisplatin-induced nephrotoxicity in a clinical context and provide better knowledge of physiological mechanisms responsible for the renoprotective effect of SAMC.
